# 

**Published:** 1893-01-14

**Authors:** 


					\32q wvrnvuKtriufi. ii^i. s>
XIII. Jan. 14th, 1893. \1L JiniL /Jr^ Monthly Farts. 6d.5 post free. 8d.
General Port Office ai a Newsiwroer for jMgt
Per Annum, 8s. 6d., post free.
|   _ Monthly Parts, 6d.; post free,
??neral Port Office ai a Newspaper for C 1 Ditto per Annum, 8s.t post free,
m the United Kingdom and Abroad.! ^
the United Kingdom and Abroad.]
IP ~
No. 329. Vol. Xm.] Satubday,
XanttabY 14th, lc93.
[Twopence.

				

